# Supplementary material for: Effects of five teaching methods in clinical nursing teaching: A protocol for systematic review and network meta-analysis
Source: PLoS One. 2022 Aug 30;17(8):e0273693. doi: 10.1371/journal.pone.0273693 (PMC9426898; doi:10.1371/journal.pone.0273693)
Supplement: S1 Table — (DOCX) [file pone.0273693.s001.docx]

| **Table 1. The initial search strategy for PubMed.** | |
| --- | --- |
| **No.** | **Search items** |
| 1 | "Students, Nursing"[Mesh] |
| 2 | ((((((Pupil Nurses[Title/Abstract]) OR (Student, Nursing[Title/Abstract])) OR (Nurses, Pupil[Title/Abstract])) OR (Nurse, Pupil[Title/Abstract])) OR (Pupil Nurse[Title/Abstract])) OR (Nursing Student[Title/Abstract])) OR (Nursing Students[Title/Abstract]) |
| 3 | #1 OR #2 |
| 4 | (((((((((((((Critical Pathway[Title/Abstract]) OR (Care Pathway[Title/Abstract])) OR (Clinical Path[Title/Abstract])) OR (Clinical Pathway[Title/Abstract])) OR (Problem-Based Learning[Title/Abstract])) OR (Problem-Based Curriculum[Title/Abstract])) OR (Problem-Based Curricula[Title/Abstract])) OR (Patient Simulation[Title/Abstract])) OR (Simulation, Patient[Title/Abstract])) OR (Case-based learning[Title/Abstract])) OR (Case-based teaching[Title/Abstract])) OR (Case method[Title/Abstract])) OR (Mentor[Title/Abstract])) OR (Mentorship[Title/Abstract]) |
| 5 | (randomized controlled trial[Publication Type]) OR (random*[Title/Abstract]) |
| 6 | #3 AND # 4 AND #14 |
